# Proteome-wide dataset supporting the study of ancient metazoan macromolecular complexes

**DOI:** 10.1016/j.dib.2015.11.062

**Published:** 2015-12-12

**Authors:** Sadhna Phanse, Cuihong Wan, Blake Borgeson, Fan Tu, Kevin Drew, Greg Clark, Xuejian Xiong, Olga Kagan, Julian Kwan, Alexandr Bezginov, Kyle Chessman, Swati Pal, Graham Cromar, Ophelia Papoulas, Zuyao Ni, Daniel R. Boutz, Snejana Stoilova, Pierre C. Havugimana, Xinghua Guo, Ramy H. Malty, Mihail Sarov, Jack Greenblatt, Mohan Babu, W. Brent Derry, Elisabeth R. Tillier, John B. Wallingford, John Parkinson, Edward M. Marcotte, Andrew Emili

**Affiliations:** a Donnelly Centre for Cellular and Biomolecular Research, University of Toronto, Toronto, Ontario, Canada; b Center for Systems and Synthetic Biology, Institute for Cellular and Molecular Biology, University of Texas at Austin, Austin, TX, USA; c Department of Medical Biophysics, Toronto, Ontario, Canada; d Department of Molecular Genetics, University of Toronto, Toronto, Ontario, Canada; e Hospital for Sick Children, Toronto, Ontario, Canada; f Department of Molecular Biosciences, University of Texas at Austin, Austin, TX, USA; g Department of Biochemistry, University of Regina, Regina, Saskatchewan, Canada; h Max Planck Institute of Molecular Cell Biology and Genetics, Dresden, Germany

**Keywords:** Proteomics, Metazoa, Protein complexes, Biochemical, Fractionation

## Abstract

Our analysis examines the conservation of multiprotein complexes among metazoa through use of high resolution biochemical fractionation and precision mass spectrometry applied to soluble cell extracts from 5 representative model organisms *Caenorhabditis elegans, Drosophila melanogaster*, *Mus musculus*, *Strongylocentrotus purpuratus,* and *Homo sapiens*. The interaction network obtained from the data was validated globally in 4 distant species (*Xenopus laevis*, *Nematostella vectensis*, *Dictyostelium discoideum*, *Saccharomyces cerevisiae*) and locally by targeted affinity-purification experiments. Here we provide details of our massive set of supporting biochemical fractionation data available *via* ProteomeXchange (PXD002319-PXD002328), PPIs *via* BioGRID (185267); and interaction network projections via (http://metazoa.med.utoronto.ca) made fully accessible to allow further exploration. The datasets here are related to the research article on metazoan macromolecular complexes in Nature [1].

**Specifications Table**

**Table:** 

Subject area	Biology
More specific subject area	Metazoan proteomics
Type of data	Set of tables
How data was acquired	Biochemical fractionation combined with quantitative mass spectrometry using LTQ XL; LTQ Orbitrap Velos
Data format	Raw and processed data
Experimental factors	• Whole body lysate from worm (*Caenorhabditis elegans*) • AX4 cells from amoeba (*Dictyostelium discoideum*) • 2 cell types in fly (*Drosophila melanogaster*), • 5 cell lines in human (*Homo sapiens*), • Embryonic stem cells from mice (*Mus musculus*) • Unfertilized sea anemone eggs (*Nematostella vectensis*) • Log-phase culture of wild type yeast W303 strain (*Saccharomyces cerevisiae*) • 5 different development stages in sea urchin (*Strongylocentrotus purpuratus*), • Stage 15–19 embryos, adult male heart and liver from frog *(Xenopus laevis)*
Experimental features	Combination of biochemical fractionation with quantitative mass spectrometry for 6387 fractions obtained from 69 different experiments, to examine the composition of soluble multiprotein complexes among diverse animal models.
Data source location	*Toronto, Canada*
Data accessibility	• Biochemical fractionations – ProteomeXchange (PXD002319-PXD002328) • PPIs – BioGRID (185267) • Complexes and interaction network projections – http://metazoa.med.utoronto.ca • MS1 and MS2 elution profiles, correlation scores and ortholog mappings – http://metazoa.med.utoronto.ca • Supplementary data with research article.

**Value of the data**

• Macromolecular complexes drive essential biological processes, yet their ubiquity across phyla is unclear. By applying a human-centric approach on the merged data for 5 species obtained through fractionation and mass spectrometry, and subsequent computational analysis we identified 16,655 high confidence protein–protein interactions and 981 putative functional modules encompassing 2153 broadly-conserved proteins found in virtually all multicellular eukaryotes.
• We further we projected a draft conservation map of >1 million putative high-confidence co-complex interactions for 122 species with fully sequenced genomes that encompasses functional modules present broadly across all extant animals.
• Functional analysis subsequently revealed metazoan-specific complexes responsible for cell–cell communication, development and disease, and ancient complexes extant for ~1 billion years with central housekeeping roles. This reconstructed physical interaction network provides mechanistic insights into the unique organization and evolution of animal cells.
• Despite the vast array of information available for many multi-cellular organisms, our data reveals fundamental attributes of the macromolecular machinery of animal cells with clear ubiquitous relevance to metazoan biology, development and evolution.
• Although our the research article focused on global conservation properties, these datasets can be analyzed at the individual animal species or complex levels by researchers in the community to assess the variety and functional adaptations of particular protein assemblies across phyla.

## 1 Data

We performed biochemical fractionation of 6387 fractions from 69 different experiments, followed by quantitative mass spectrometry analysis to derive soluble multiprotein complexes from 5 representative model organisms with 4 other organisms used for validation. Altogether the selected organisms cover a span of over half a billion years of evolutionary divergence from human, and also play a vital role as model organisms in biology and disease research. We selected 5 human cell lines, 2 cell types in fly, and cells from 5 different development stages in sea urchin, along with whole body lysate from worm and embryonic stem cells from mice. These 5 species were used in deriving interactions and complexes, while 4 additional species were subjected to the same preparation and quantitation methods, but used only in validation: frog, sea anemone, yeast and amoeba. The species, cell types, and fractionation methods used are provided as Supplementary information with the research article.

## 2 Experimental design, materials and methods

### 2.1 Sample Preparation

#### 2.1.1 C. elegans

L4 stage N2 worms were suspended in lysis buffer (10 mM HEPES pH 7.9, 1.5 mM MgCl_2_, 10 mM KCl) that contained freshly added EDTA-free protease (Complete-Mini; Roche) as well as phosphatase (PhosSTOP; Roche) inhibitor cocktail (1 tablet each per 10 ml), then lysed by 3×10 s sonication on ice using a Sonifier 450 (Branson, output 6.0, duty cycle 60%). Protein lysates were clarified by centrifugation, concentration measured by Bradford assay. Affinity bead (SeraFILE PROspector) based sample pre-separations were performed as per manufacturer׳s instructions.

#### 2.1.2 D. discoideum

One liter of AX4 cells were grown in HL5 medium, harvested at a cell density of 4–5×10^6^ cells/ml, transferred to 17 mM phosphate buffer for 2 h, pelleted and frozen in aliquots of 5×10^8^ cells each. Cell pellets were resuspended in lysis buffer and lysed by sonication as mentioned above. Prior to biochemical fractionation, removal of nucleic acids was done by treating soluble protein extracts with benzonase nuclease (100 µ/ml; Millipore, USA) on ice for 30 min.

#### 2.1.3 D. melanogaster

Nuclear extracts of SL2 cells [5], [6] and whole cell extracts were prepared by harvesting cells using centrifugation at 1200 rpm for 10 min at 4 °C, removing medium by aspiration, and washing cells twice with cold PBS. Cell pellets were resuspended in lysis buffer (20 mM Hepes/KOH pH 7.6, 200 mM KCl, 10% Glycerol, 0.1% NP-40, 1 mM DTT, PMSF, Aprotinin, Leupeptin and Pepstatin) and incubated on ice for 5 min. Cells were frozen in liquid nitrogen and thawed in 26 °C water bath three times to lyse, and the extract clarified at 13,000 rpm for 30 min at 4 °C, before being aliquoted and snap-frozen at −80 °C for subsequent analysis.

#### 2.1.4 H. sapiens

Human neural stem cell line CB660 and human brain tumor stem cell line G166 were obtained from Patrick Paddison (Fred Hutchinson Cancer Research Center, Seattle). Cell pellet was resuspended in lysis buffer [10 mM Tris–HCl (pH 8.0), 10 mM KCl, 1.5 mM MgCl_2_, 0.5 mM DTT, and 1x Protease Inhibitor Cocktail Set I (Calbiochem)], centrifuged at 1000*g* for 5 min (4 °C). The supernatant was saved as the cytosolic fraction. The pellet was resuspended in 250 mM sucrose/10 mM MgCl_2_/1x Protease Inhibitor Cocktail, layered over a sucrose cushion of 880 mM sucrose/0.5 mM MgCl_2_/1x Protease Inhibitor Cocktail, and centrifuged at 3000*g* for 10 min (4 °C). The pellet was resuspended in lysis buffer with 5% NP-40 by sonicating water bath (15 min) followed by centrifugation at 3500*g* for 10 min and the supernatant saved as the nuclear fraction [2].

#### 2.1.5 M. musculus

Brachyury-GFP tagged mouse embryonic stem cells [7] were maintained in DMEM/F12 and Neurobasal buffer (Invitrogen) with addition of N2-Supplement, B27+RA, 10% bovine serum albumin (BSA), 2 mM GlutaMAX, 100 units/ml penicillin, 100 μg/ml streptomycin, 1.5×10^−4^ M monothioglycerol (MTG, Sigma), LIF (1%) and BMP4 (10 ng/ml). ES cells were cultured on gelatin coated tissue culture polystyrene and dissociated with TrypLE (Invitrogen). Harvesting of cells was done using 10% fetal bovine serum (FBS) in DMEM and centrifuged at 900*g* for 5 min. The cell pellet was washed with IMDM and centrifuged at 900*g*, then resuspended and lysed with the same protocol as for human cells [2].

#### 2.1.6 N. vectensis

Unfertilized sea anemone eggs samples were collected by centrifugation, aspirating away seawater by vacuum. Samples were resuspended in 5 volumes of ice-cold lysis buffer and wash three times. (Lysis buffer: 40 mM NaCl, 2.5 mM MgCl_2_, 300 mM glycine, 100 mM potassium gluconate, 2% glycerol, 50 mM HEPES and pH 6.9 4.19 mM CaCl_2_, 10 mM EGTA) supplemented with fresh protease and phosphatase inhibitors (1 μΜ PEFABLOC, 10 μM Protease inhibitor Cocktail 3 Cal Biochem, 1 mM Na orthovanadate, 100 μM NaF). Suspensions were transferred to a chilled glass homogenizer on ice, allowed to settle; buffer removed, and then disrupted by hand using 5–10 strokes of a loose fitting pestle on ice until 100% lysis was obtained. Lysates were centrifuged at 10,000*g* for 15 min at 4 °C, and the clarified supernatants removed for analysis.

#### 2.1.7 S. purpuratus

Four stages of sea urchin early embryonic development were analyzed: unfertilized embryos, 5 min post fertilization, 2 cell and hatched blastula. Samples were collected by centrifugation, aspirating away seawater by vacuum. Samples were resuspended in 5 volumes of ice-cold lysis buffer and washed three times. (Lysis buffer: 40 mM NaCl, 2.5 mM MgCl_2_, 300 mM glycine, 100 mM potassium gluconate, 2% glycerol, 50 mM HEPES with pH 6.9 4.19 mM CaCl_2_, 10 mM EGTA) for unfertilized eggs and pH 7.4 (8.56 mM CaCl_2_, 10 mM EGTA) for fertilized embyros with KOH, and freshly added protease and phosphatase inhibitors (1 mM PEFABLOC, 10 mM Protease inhibitor Cocktail 3 Cal Biochem, 1 mM Na orthovanadate, 100 mM NaF). Suspensions were transferred to a chilled glass homogenizer on ice, allowed to settle; buffer removed, and then disrupted by hand using 5–10 strokes of a loose fitting pestle on ice until 100% lysis was obtained. Lysates were centrifuged at 10,000*g* for 15 min at 4 °C, and the clarified supernatants removed for analysis. Protein concentrations were measured by Bradford assay. Affinity bead (SeraFILE PROspector) based sample pre-separations were performed as per manufacturer׳s instructions.

#### 2.1.8 S. cerevisiae

50 ml of a log-phase culture of wild-type W303 strain yeast (A600=1.0) were centrifuged, the cells washed in 1 ml H_2_O, resuspended in 200–300 μl modified extraction buffer (50 mM HEPES pH 7.8, 200 mM KCl, 1 mM Na2EDTA, 5 mM EGTA-KOH, 10% glycerol, 1 mM DTT) with protease inhibitor cocktail (Roche Diagnostics, Mannheim, Germany), and lysed using a bead beater with 0.4 ml glass beads. The lysate was centrifuged at 14,000 rpm for 5 min and the supernatant (clarified whole cell extract).

The above lysates or fractions from beads for all species except frog were subjected to ion exchange fractionation by an Agilent 1100 HPLC system. Proteins from each of the various HPLC fractions were precipitated, resuspended and digested in solution with trypsin, dried and re-solubilised [2] before being analyzed by LC–MS/MS using a nanoflow HPLC System (EASY-nLC; Proxeon) coupled with LTQ Orbitrap Velos (Thermo Fisher).

#### 2.1.9 X. laevis

Extracts were prepared from 750 stage 15 embryos, from 1000 dissected animal caps allowed to develop to stage 19–20, or from adult male heart and liver. All steps were on ice or 4 °C unless otherwise noted. Embryos were washed in X Buffer (10 mM Tris pH 7.5, 20 mM KCl, 5 mM MgCl_2_, with 50 µg/ml cycloheximide and 1:100 volume of Protease Inhibitor Cocktail Set I (Calbiochem) added freshly), and glass dounce homogenized in an equal volume of X Buffer using 20 strokes of loose and tight pestles. After 1000*g* 10 min initial centrifugation, the supernatant was further clarified by re-centrifugation at 15,000*g* 10 min. Animal caps were washed in Steiner׳s medium, liquid was removed, and tissue stored −80 °C until use. After dounce homogenization, sample was probe-tip sonicated with 2 pulses (30 s 30% power) prior to clarification centrifugation as above. Heart and liver were dissected from one frog, minced with a razor blade, disrupted with a Tissue Tearor (BioSpec Products) in an equal volume of X Buffer, glass dounce homogenized with a loose pestle, and large debris was pelleted 1000*g* 1 min. The supernatant was dounced with a tight pestle and then clarified as for embryo extract. In a replicate experiment, clarified heart and liver homogenates were frozen, and subsequently pooled, depleted of hemoglobin using HemogloBind (Biotech Support Group) according to the manufacturer׳s instructions, and clarified at 15,000*g* 10 min prior to sucrose gradient fractionation. Protein concentration was determined with BioRad Protein Assay, using BSA as a standard. Gradients were formed by layering 3.5 ml/3.9 ml/3.9 ml respectively of 47/26.5/7% sucrose in X Buffer without protease inhibitors and were allowed to equilibrate during horizontal storage at 4 °C for 1.5–3 h prior to loading. [Gradients used in fractionating *Xenopus laevis* heart/liver homogenate containing hemoglobin underwent minor mixing.] After loading 200–500 µl extract (containing 1.8 mg embryo, 0.6 mg animal cap, 2.5 mg each pooled heart/liver extract, or 2 mg hemoglobin-depleted pooled heart/liver) the gradients were centrifuged 35,000 rpm 1.5 h 4 °C in an SW41 rotor with braking to 800 rpm. Fractions were collected by volume displacement through a UV flow cell monitoring absorbance at 254 nm. Proteins were precipitated with trichloroacetic acid, or (for more dilute animal cap gradient fractions) UPPA-Protein-Concentrate (G Biosciences), and washed with ice cold acetone, and the air dried pellets resuspended in 0.1 M Tris pH 8.1 for in-solution digestion with trypsin, then analysis with LTQ Orbitrap Velos (Thermo Fisher).

## 3 Data processing protocol

Target-decoy databases were constructed from protein sequences downloaded from ENSEMBL when available (*Homo sapiens*, *Mus musculus*, *Caenorhabditis elegans*, *Drosophila melanogaster, Saccharomyces cerevisiae*), and otherwise from databases of the main species-specific genomics community (*Strongylocentrotus purpuratus*: spbase.org, *Dictyostelium discoideum*: disctybase.org, *Nematostella vectensis*: genome.jgi-psf.org and *X. laevis*: http://www.marcottelab.org/index.php/Xenopus_Genome_Project) and processed to retain only the longest sequence for each gene, to simplify orthology mapping between species, since determination of conserved complexes was the primary focus of the project.

To improve peptide-spectral matching sensitivity and accuracy in obtaining MS2 peptide identification and spectral count quantitation mass spectra were searched using 3 search engines: Tide, INSPECT and MSGFDB each employing a different search methodology, and the spectral counts were integrated probabilistically using MSblender [3]. We found we were able to increase the total peptide-spectral matches and proteins identified by 20–60% depending on the sample compared to using Sequest alone, with a false discovery rate of <1% for each sample. To eliminate spurious associations between proteins with high sequence similarity, such as in the case of close homologs, only unique peptides were retained. Tide search output is in a format that was processed for best hits with MSblender; MSGFDB and INSPECT search output is provided directly, when available. The result was a total of 10.2 million peptide-spectral matches from the 5 species integrated into the metazoan complex map. The mass spectrometry search output files are available for download from ProteomeXchange.

## 4 MS1 and MS2 protein identification and quantitation

We further used MS1 intensities as a means of improving the accuracy of protein quantitation with PepQuant35 [4]. To prepare cleaner protein count profiles, we filtered the protein quantitation to retain only proteins identified previously in a given sample using the MS2 spectral count methods described in the preceding paragraph.

The MS1 intensity and MS2 spectral count elution profiles were used to derive four different correlation scores for given protein pair: (1) Pearson correlation with added Poisson noise, (2) weighted cross – correlation, (3) co-apex score, all from MS2 and (4) Euclidean distance from MS1 using the open-source SciPy python library [8]. The correlation profiles for the four test species were mapped back to their human orthologs. These scores along with external biochemical [9], [10] and functional evidence [11] were used as input for machine learning to predict conserved protein–protein interactions and complex co-memberships.

The MS1 and MS2 elution profiles, correlation scores and orthology mapping files used are available for download from the supporting website http://metazoa.med.utoronto.ca.

## Footnotes

Appendix A Supplementary data associated with this article can be found in the online version at doi:10.1016/j.dib.2015.11.062.
